# Deep Brain Stimulation of Hemiparkinsonian Rats with Unipolar and Bipolar Electrodes for up to 6 Weeks: Behavioral Testing of Freely Moving Animals

**DOI:** 10.1155/2017/5693589

**Published:** 2017-07-03

**Authors:** Kathrin Badstuebner, Ulrike Gimsa, Immo Weber, Armin Tuchscherer, Jan Gimsa

**Affiliations:** ^1^Department of Neurology, University of Rostock, Rostock, Germany; ^2^Institute of Behavioural Physiology, Leibniz Institute for Farm Animal Biology, Dummerstorf, Germany; ^3^Institute of Genetics and Biometry, Leibniz Institute for Farm Animal Biology, Dummerstorf, Germany; ^4^Chair of Biophysics, University of Rostock, Rostock, Germany

## Abstract

Although the clinical use of deep brain stimulation (DBS) is increasing, its basic mechanisms of action are still poorly understood. Platinum/iridium electrodes were inserted into the subthalamic nucleus of rats with unilateral 6-OHDA-induced lesions of the medial forebrain bundle. Six behavioral parameters were compared with respect to their potential to detect DBS effects. Locomotor function was quantified by (i) apomorphine-induced rotation, (ii) initiation time, (iii) the number of adjusting steps in the stepping test, and (iv) the total migration distance in the open field test. Sensorimotor neglect and anxiety were quantified by (v) the retrieval bias in the corridor test and (vi) the ratio of migration distance in the center versus in the periphery in the open field test, respectively. In our setup, unipolar stimulation was found to be more efficient than bipolar stimulation for achieving beneficial long-term DBS effects. Performance in the apomorphine-induced rotation test showed no improvement after 6 weeks. DBS reduced the initiation time of the contralateral paw in the stepping test after 3 weeks of DBS followed by 3 weeks without DBS. Similarly, sensorimotor neglect was improved. The latter two parameters were found to be most appropriate for judging therapeutic DBS effects.

## 1. Introduction

Electrical stimulation of the brain is an emerging area for the treatment of a growing number of neurological and psychiatric diseases. Deep brain stimulation (DBS) is well established for the treatment of movement disorders, such as Parkinson's disease (PD) [1]. For patients in the advanced stages of PD, DBS of the* subthalamic nucleus* (STN) is highly effective in reversing motor deficits. In addition to locomotor and sensorimotor deficits, PD patients also suffer from emotional disturbances, namely, depression and anxiety. Anxiety may result not only from the impairment of motor function but also from dysfunction in the STN [2]. More recently, DBS has also been applied at earlier stages of PD [3]. Nevertheless, STN-DBS does not always improve symptoms, and it may actually worsen them [4]. However, only limited information is available on (i) the effects of DBS on cognitive and emotional traits; (ii) the efficiency of different stimulation modes, in particular unipolar versus bipolar stimulation; and (iii) the long-term sustainability of symptom alleviation after the cessation of DBS.

The STN is one of the most important target regions for high-frequency (approx. 130 Hz) DBS in patients, especially in patients in the advanced stages of PD who are refractory to conventional therapy [5, 6]. Historically, DBS was developed as a modification of ablative surgery, in which basal ganglia, such as the STN, were irreversibly destroyed as a final treatment option in late-stage PD [7]. During surgery, electric stimulation was used to guide neurosurgeons to the precise position of the lesions. The main advantages of DBS over surgical lesions are its reversibility and the ability to modulate the stimulation parameters [8]. It has been well documented that DBS of the STN may also improve the cardinal motor symptoms of PD in the long-term [9, 10].

Maesawa et al. [11] were the first to describe a DBS-related protection of dopaminergic neurons in the SNc by STN-DBS of 6-OHDA hemiparkinsonian rats. Later, Harnack et al. [12] described a preservation of approx. 50% of the dopaminergic nigral neurons in the SNc by STN-DBS compared to sham-stimulated and naïve rats. Spieles-Engemann et al. [13] demonstrated an increase in the levels of the brain-derived neurotrophic factor and Wu et al. [14] observed decreased apoptosis in the nigrostriatal system after STN-DBS of 6-OHDA-lesioned rats. Other authors have described the preservation of neurons or even neurogenesis by DBS in other brain regions [15, 16].

Clearly, more information is needed to explore the full therapeutic potential of DBS. For example, optimum target regions are not always known, and the basic mechanisms by which DBS acts are still poorly understood [17–22]. In addition, adverse side effects cannot always be avoided. Therefore, research on both the optimal DBS technique and its neurological mechanisms is needed. To allow for a comparison with the clinical situation, the availability of animal models for long-term examinations and behavioral testing is of the utmost importance. Many groups have reported results from animal models with external stimulators, although these used very short durations of DBS. In some cases, only anaesthetized animals were used. Long-term behavioral outcomes have not been sufficiently examined (for reviews see [23, 24]). Nevertheless, miniaturized mobile stimulators for the chronic instrumentation of freely moving mice or rats for up to five weeks have recently been developed by a few groups [11, 25–32], including our own [33]. Such animal models allow the testing of drug-induced or spontaneous behaviors as a way to quantify the effects of lesion-induced or DBS-induced changes in locomotor function and behavior.

In pioneering work on experimental DBS, stainless steel electrodes have been used to optimize the electrode position in the brain [34, 35]. However, stainless steel electrodes are obsolete and not ideal for current studies. Their use in long-term experiments is prevented because of corrosion and the detrimental effects this has on the surrounding brain tissue [36–38].

Here, we combined a revised version of our miniaturized constant-current-pulse generator [33] with new Pt/Ir electrodes to test the effects of different modes of STN-DBS on the behavioral performance of 6-OHDA-induced hemiparkinsonian rats [39]. Several tests have been developed for the detailed evaluation of spontaneous motor and sensorimotor function in rodents [40–42]. Here, we chose four different behavioral tests: (i) an apomorphine-induced rotation test [43, 44], (ii) the stepping test [45], (iii) the corridor test [46], and (iv) a modified version of the classical open field test [47]. From these tests, six quantitative parameters were determined to describe the effects of lesion- and DBS-induced changes in locomotor function, sensorimotor neglect, exploration, and anxiety-like behavior.

## 2. Materials and Methods

### 2.1. Animals

Male Wistar Han rats (240 g–260 g; Crl:WI(Han) Rattus norvegicus: RRID:RGD_2308816) were obtained from Charles River Laboratory (Sulzfeld, Germany) and housed under temperature-controlled conditions in a 12-h light-dark cycle with conventional rodent chow and water provided ad libitum. The study was carried out in accordance with the European Community Council directive 86/609/EEC for the care of laboratory animals and was approved by the local animal care committee (LALLF M-V/TSEM/7221.3-1.2-019/10).

### 2.2. Electrodes

**Figure 1:**
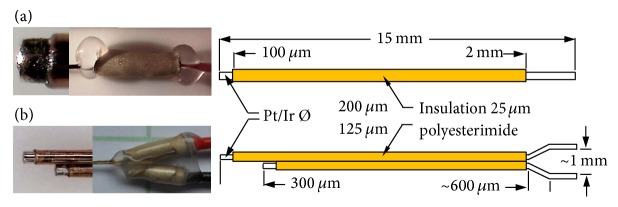
Photographs of tips (left), distal connections (center), and schematic drawings (right) of (a) the unipolar (200 *µ*m wire diameter) and (b) the bipolar microelectrode (125 *µ*m wire diameter). The electrode shafts were insulated with 25 *µ*m polyesterimide. All electrode tips were bare for 100 *µ*m.

Two types of microelectrodes were custom-made from round Pt/Ir alloy (Pt90/Ir10) wires, which were insulated with polyesterimide but left bare at the tips (Figure 1). The unipolar microelectrodes were purchased from Polyfil (Zug, Switzerland) and the bipolar microelectrodes were purchased from FHC (Bowdoin, ME, USA). Their distal ends were connected with biocompatible insulated wire. To avoid excessive heating from soldering, the cables were connected with conductive silver glue, covered by biocompatible heat-shrink tubing and sealed with biocompatible silicon glue (NuSil Technology, Carpinteria, USA). The unipolar electrodes were driven against a gold-wire counter electrode (length 30 mm, diameter 200 *µ*m). The bipolar electrodes did not require the implantation of an additional counter electrode.

### 2.3. Surgery

The surgical procedures were performed using a stereotactic frame (Stoelting, Wood Dale, IL, USA). Rats were anesthetized by intraperitoneal injection of ketamine hydrochloride (10 mg per 100 g body weight, Ketanest S®, Pfizer, Karlsruhe, Germany) and xylazine (0.5 mg per 100 g body weight, Rompun®, Pfizer). During surgery, their eyes were protected from dehydration by Vidisic® (Bausch and Lomb, Berlin, Germany).

The skull was opened using a dental rose-head bur (Kaniedenta, Herford, Germany). To induce hemiparkinsonism, rats were lesioned with a unilateral injection of 6-OHDA into the right medial forebrain bundle. Twenty-four *µ*g 6-OHDA dissolved in 4 *µ*l 0.1 M citrate buffer were delivered over 4 min via a 5 *µ*l Hamilton microsyringe. Sham-lesioned rats received 4 *µ*l 0.1 M citrate buffer delivered in the same fashion. The stereotactic coordinates, relative to bregma, were anterior-posterior (AP: −2.3 mm), medial-lateral (ML: 1.5 mm), and dorsal-ventral (DV: −8.5 mm) ([48]; RRID:SCR_006369). After surgery, the wound was sutured and the animals received 0.1 ml novaminsulfone (Ratiopharm, Ulm, Germany) and 4 ml saline subcutaneously. To prevent hypothermia, a heat lamp was used until vital functions returned to normal. The success of the lesion procedure was evaluated with the apomorphine-induced rotation test 12–14 days after surgery.

**Figure 2:**
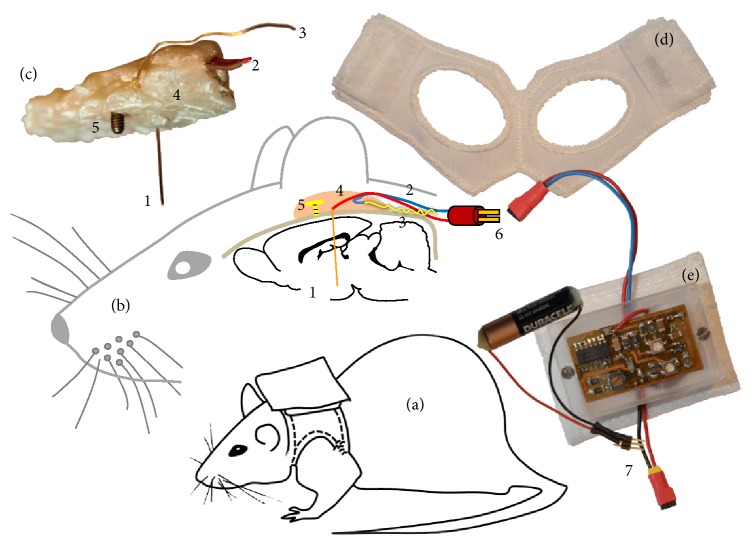
Schematic views of the unipolar DBS rat model. (a) Rat with stimulator in backpack; (b) sagittal view illustrating the locations of the implanted unipolar DBS electrode; (c) image of an explanted DBS mounting; (d) backpack vest with Velcro hooks; (e) stimulator in PMMA housing with pocket and current-pulse battery. 1: unipolar Pt/Ir electrode; 2: electrode cables; 3: gold-wire counter electrode; 4: biocompatible dental acrylic embedding all components; 5: anchor screw to fix the acrylic mounting to the skull; 6: electrode connector; 7: current-pulse battery connector.

Approximately 3 weeks after lesion induction, the electrodes were implanted with their stimulating tips localized in the STN. The tip coordinates, relative to bregma, were AP: −3.5 mm, ML: 2.4 mm, and DV: −7.6 mm ([48]; RRID:SCR_006369). The shorter counter electrode tips of the bipolar electrodes were oriented so that they were lateral to the stimulating tip. The electrode shafts were fixed to the skull by an adhesive-glue bridge of dental acrylic resin (Pontiform automix 10 : 1, Müller & Weygandt GmbH, Büdingen) attached to an anchor screw fixed to the skull above the left hemisphere.  Figure 2 illustrates the unipolar electrode orientation.

**Figure 3:**
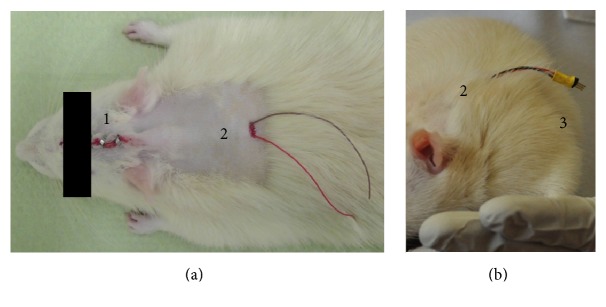
(a) Details of the outlet port for the subcutaneous cables centered at the dorsum. (b) Rat with electrode connector one week after surgery. 1: suture clips; 2: dorsal cable outlet port; 3: crimped plug connector.

Following electrode implantation, the cables of the stimulating and the counter electrode contacts were implanted subcutaneously with a central dorsal outlet port (Figure 3(a)). After surgery, the rats were treated in the same manner as after the 6-OHDA injection. Rats were allowed to recover for eight days before stimulation started.

### 2.4. Chronic Instrumentation

One week after surgery, a plug connector (M52-040023V0545, Harwin Plc, Hampshire, UK) was crimped to the electrode cables (Figure 3(b)). The connector ensured flexibility in the use of commercial rat jackets (Lomir Biomedical, Quebec, Canada), which contained the stimulators and batteries in a custom-made fabric backpack (Figure 2(e)).

The setup allowed for the completely free movement of the animals over long periods of time. The stimulator plate was protected from mechanical strain and moisture by a custom-made polymethyl-methacrylate box and was connected to the external current-pulse battery. At the start of the stimulation, the electrode connector was plugged into the stimulator (Figure 2). The entire stimulator system was miniaturized and designed for minimum power consumption relative to our preliminary versions [33]. A separate long-lasting pulse-generator battery was inserted at the bottom of the DBS stimulator. Only the current-pulse battery (Figure 2(e)) had to be exchanged at 4 weeks.

The jacket and cables were checked daily to ensure the long-term effectiveness of the device. Cables that were torn off by the animal in exceptional cases were replaced immediately. The jackets had to be replaced every week because of wear. The stimulator signal was checked with an oscilloscope at the same time as the jackets were replaced. There has never been a problem with the batteries or the stimulator hardware.

### 2.5. Stimulation Conditions

The stimulator provided rectangular monophasic current pulses. Different treatment groups were stimulated for 3 days, 3 weeks, or 6 weeks. In all experiments, the stimulators were adjusted to a pulse width of 60 *µ*sec with the negative pulse current of −200 *µ*A applied to the stimulating unipolar electrode or to the proximal tip of the bipolar electrode. The pulse repetition frequency was 130 Hz. For sham-DBS controls, only bipolar electrodes were used because they induce more mechanical stress to the tissue during the surgical procedure.

### 2.6. Behavioral Tests

**Figure 4:**
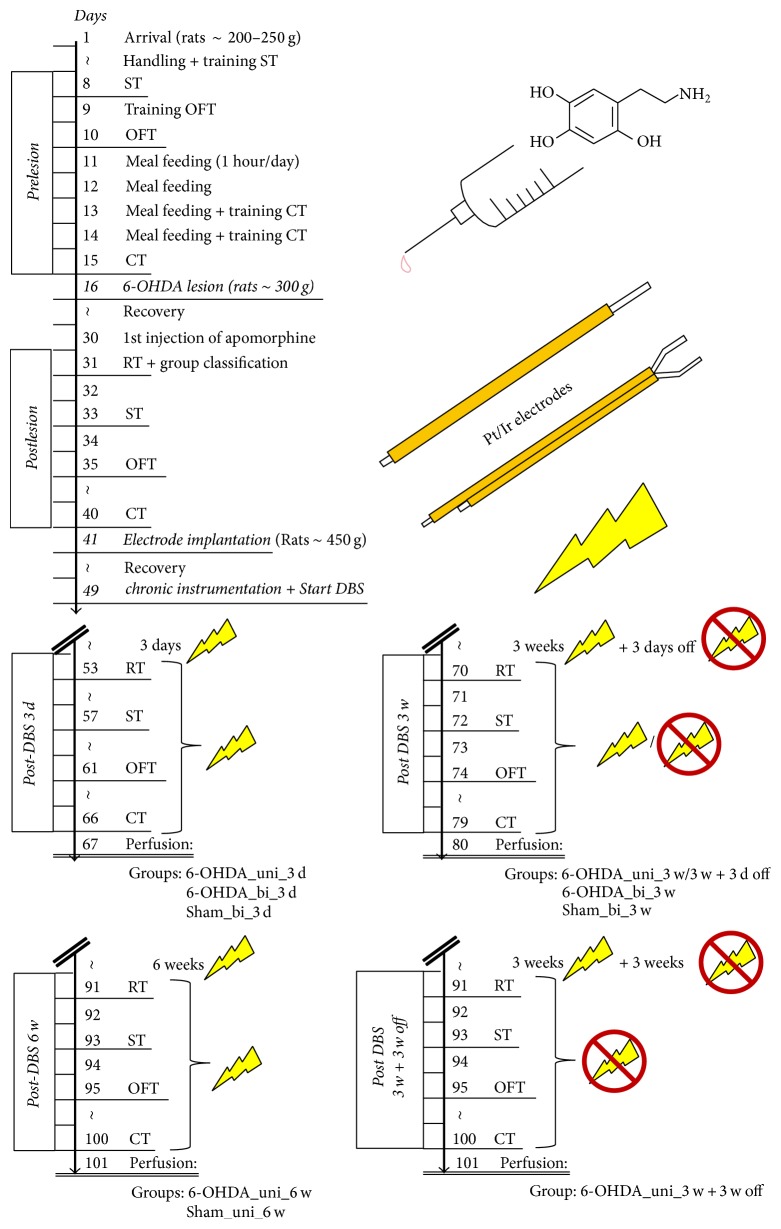
Schedules of the test procedures. RT, ST, OFT, and CT refer to the rotation, stepping, open field, and corridor tests, respectively.

The effects of lesion- and DBS-induced changes in the animals' behavior were quantified using the drug-induced apomorphine-stimulated rotation test and three non-drug-induced tests (the stepping, corridor, and open field tests). Experiments were conducted at different times: (i) prior to lesion induction; (ii) 12–14 days after lesion or sham lesion induction; (iii) after 3 days of DBS or 3 days with the stimulator off (sham stimulation); (iv) after 3 weeks of DBS or 3 weeks with the stimulator off (sham stimulation); (v) ≥3 days after the cessation of DBS subsequent to 3 weeks of DBS; (vi) after 6 weeks of DBS; and (vii) 3 weeks after the cessation of DBS subsequent to 3 weeks of DBS. For details see Table 1 and Figure 4.

### 2.7. Apomorphine-Induced Rotation Test

For assessing drug-induced locomotor function, apomorphine (0.25 mg/kg body weight dissolved in saline) was injected subcutaneously. Rotation was quantified in a custom-made “rodent-rotometer” modified according to Ungerstedt and Arbuthnott [44]. The rate of pathological circling, in rotations per minute (rpm), contralateral to the 6-OHDA-lesion site was determined electronically over 40 min. Rotation counts of at least 2 rpm indicated successful lesions. Subsequently, animals were assigned to groups such that the groups were composed of rats that had approximately the same mean rotation values. The rotation tests were repeated after one day because the first apomorphine application did not usually result in the maximum response.

### 2.8. Stepping Test

The stepping test, which assesses forelimb akinesia, was essentially performed as described by Olsson et al. [45]. In brief, rats were set on a table and allowed to settle with all limbs on the table. The experimenter then lifted the lower body by grabbing the rat around neck and behind the forepaws in a way that only the forepaws were touching the table. Then, one forepaw was restrained, and the free forepaw, which was still touching the table, was moved sideways at a steady pace along the table surface in both directions at a rate of approximately 1 m per 5 s. The number of adjusting steps was counted manually for the movement of both forepaws in the forehand and backhand directions. For both directions, the contralateral bias was calculated as the percentage of steps of the contralateral paw with respect to the sum of the steps of both forepaws (50% was expected for control). In addition, the time needed for the initiation of the first adjusting step toward the rats' home cage was recorded for both forepaws. In the cases of an immediate response (i.e., when the initiation time was too short to be registered manually), the time was recorded as zero.

### 2.9. Corridor Test

To assess sensorimotor neglect, we used the corridor test [46]. A long narrow wooden corridor (240 cm long, 7 cm wide, and 23 cm high) was equipped with 14 equidistant pairs of adjacent pots (diameter: 1.2 cm) placed along both sides of the corridor. Each pot contained five sucrose reward tablets (5TUT; TestDiet®, USA). The clear plastic lid of the corridor allowed us to observe the rats during testing. Before the tests, rats were food-restricted with only 1 hour per day allowed for feeding (“meal feeding”) for 4 days. Two tests were performed on two consecutive days under the same conditions. The trials started with placing the rat into one end of the corridor, where it was free to explore, turn around, and feed on pellets. One “retrieval” was counted when the animal poked its nose into a pot with sugar pellets, regardless of whether it actually retrieved or fed on any pellets. The exploration of each new pot was counted as an additional retrieval. To reduce exploration behavior of the corridor itself, rats were placed into an identical, empty corridor beforehand. The number of retrievals ipsilateral and contralateral to the side of the lesion were recorded manually over 5 min. The contralateral bias was expressed as the percentage of the retrievals made on the contralateral side relative to the total number of retrievals.

### 2.10. Open Field Test

Spontaneous mobility and anxiety were evaluated by placing the rats in a square open field arena (46 cm × 45 cm) inside an isolation box. The animals were kept in the dark in the examination room 1 h before the start of the test. The open field was illuminated by a white photo bulb providing 200 to 250 Lux. During testing, rats were monitored by a video camera. The open field was divided into a center area (22 cm × 22 cm) and a peripheral zone using the tracking software Ethovision XT (Noldus Information Technology, Leesburg, VA, USA; RRID:SCR_000441). This allowed for the automatic recording of the rat's movement in the two zones. Each rat was tested once for 10 minutes. After each session, the open field was cleaned to prevent odor from influencing the next animal's behavior. The total migration distances were taken as a measure of spontaneous mobility and the ratio of the migration distance within the center area to the total distance moved was interpreted as a measure of anxiety.

### 2.11. Statistics

Data analysis was conducted with the SAS software package, Version 9.4 for Windows (Copyright, SAS Institute Inc., Cary, NC, USA, RRID:SCR_008567). Descriptive statistics and tests for normality were calculated with the UNIVARIATE procedure using Base SAS software. Data that could be considered as approximately normal was analyzed by one-way repeated measurement ANOVA with the MIXED procedure of the SAS/STAT software module. The models for the investigated treatments contained the fixed factor “time” with different levels (prelesion, postlesion, 3 d, 3 w, 3 w + 3 d, and 6 w) for each treatment. Repeated measures on the same animal were taken into account in the REPEATED statement of the MIXED procedure using time as the repeated effect, the SUBJECT = animal option to define the blocks of the residual covariance matrix and the TYPE = CS option to define their covariance structure. Least-square means (LSM) and their standard errors (SE) were computed for each time level of each treatment and compared with the “postlesion”-LSM using the Dunnett-Hsu procedure (pairwise multiple comparisons with the control).

The investigated treatments for each time (prelesion, postlesion, 3 d, 3 w, 3 w + 3 d, and 6 w) were analyzed by one-way ANOVA with the MIXED procedure of the SAS/STAT software module. The models for the times contained the fixed factor treatment (see Table 1). LSM and their SE were computed for each treatment level of each time and were compared pairwise using the Tukey-Kramer procedure (pairwise multiple comparisons of all possible pairs). Effects and differences were considered significant for *p* ≤ 0.05.

## 3. Results

### 3.1. Confirmation of Electrode Placement

The localization of electrode tips in the STN was evaluated by retrospective analyses of Nissl-stained cryosections of the STN of selected rats. It suggested a precise electrode placement in approximately 75% of the cases, analogous to the success rate of the lesion surgery (see below). A comprehensive histological evaluation is currently underway.

### 3.2. Locomotor Activity

**Figure 5:**
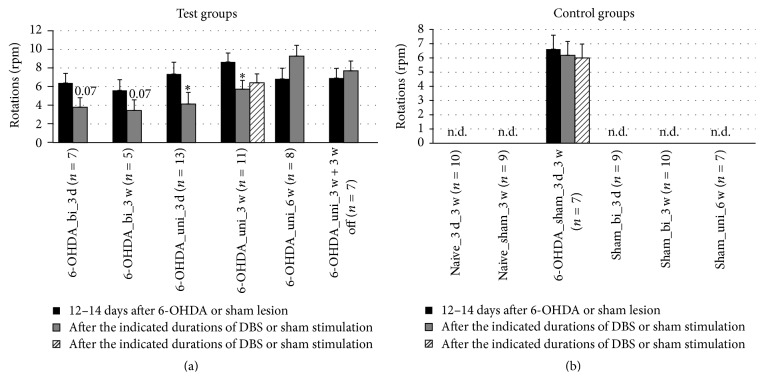
Short-term and long-term effects of DBS with uni- and bipolar electrodes on the apomorphine-induced rotation behavior of hemiparkinsonian rats. (a) and (b) refer to test groups and controls, respectively. Different column patterns indicate the different times of behavioral testing; black: 12–14 days after 6-OHDA or sham lesion; gray and hatched: after the durations of DBS or sham stimulation indicated in the group labels. For experimental details see Table 1. Significance levels are indicated with asterisks as follows: ^*∗*^*p* ≤ 0.05, b (borderline): 0.05 < *p* ≤ 0.08 according to one-way ANOVA. Asterisks refer to comparisons with corresponding black columns.

The success of lesion induction was evaluated based on the apomorphine-induced rotation test results 12–14 days after surgery. The success rate (rpm ≥ 2) was approximately 75%, and the mortality rate was less than 10%. In the apomorphine-induced rotation test, reduced rotation was detected with DBS after 3 days and after 3 weeks. In these cases, unipolar stimulation was more effective than bipolar stimulation (Figure 5). However, after 6 weeks, pathological rotation was detected again, regardless of whether the DBS had been continued or discontinued after 3 weeks. As expected, no pathological rotation was detected in naïve or sham-lesioned rats.

Spontaneous locomotor activity was assessed based on the total migration distance in the open field test. 6-OHDA lesions reduced the total migration distance, whereas naïve rats showed a marginal increase in total distance with each trial, which can be explained by habituation to the open field with repeated exposure. In contrast, DBS reduced the total migration distance, in most of the groups. The total migration distance increased at 3 days after the cessation of DBS subsequent to 3 weeks of DBS with unipolar electrodes (Figure 11).

### 3.3. Akinesia

**Figure 6:**
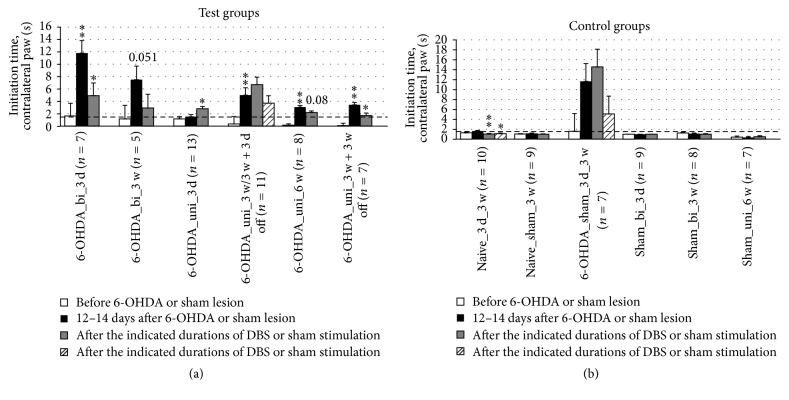
Short-term and long-term effects of DBS with uni- and bipolar electrodes on akinesia as measured by the initiation time of the first adjusting step of the contralateral forepaw in the stepping test. (a) and (b) refer to test groups and controls, respectively. The dashed line at 1 s allows for an easier comparison with Figure 7 and between (a) and (b). Different column patterns indicate different times of behavioral testing; white: before 6-OHDA or sham lesion; black: 12–14 days after 6-OHDA or sham lesion; gray and hatched: after the durations of DBS or sham stimulation indicated in the group labels. For experimental details see Table 1. Significance levels are indicated with asterisks as follows: ^*∗∗*^*p* ≤ 0.01, ^*∗*^*p* ≤ 0.05, b (borderline): 0.05 < *p* ≤ 0.08 according to one-way ANOVA. Asterisks above black columns refer to white columns; all others refer to black columns.

To assess the effects of the lesion-induced akinesia, the parameters “initiation time of paw movement” and “number of adjusting steps” were recorded in the stepping test. In rats receiving bipolar DBS, a significant reduction in the initiation time of contralateral forepaw stepping was observed after 3 days but not after 3 weeks of DBS (Figure 6). In long-term, unipolar DBS, significant improvements were found 3 weeks after the cessation of DBS subsequent to 3 weeks of DBS. A similar effect was observed after 6 weeks of continuous stimulation, although with borderline significance.

**Figure 7:**
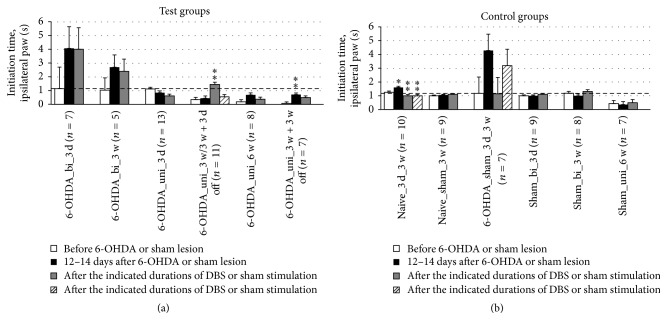
Short-term and long-term effects of DBS with uni- and bipolar electrodes on the akinesia of hemiparkinsonian rats as measured by the initiation time of the first adjusting step of the ipsilateral forepaw in the stepping test. (a) and (b) refer to test groups and controls, respectively. The dashed line at 1 s allows for an easier comparison with Figure 6 and between (a) and (b). Different column patterns indicate different times of behavioral testing; white: before 6-OHDA or sham lesion; black: 12–14 days after 6-OHDA or sham lesion; gray and hatched: after the durations of DBS or sham stimulation indicated in the group labels. For experimental details see Table 1. Significance levels are indicated with asterisks as follows: ^*∗∗*^*p* ≤ 0.01, ^*∗*^*p* ≤ 0.05, b (borderline): 0.05 < *p* ≤ 0.08 according to one-way ANOVA. Asterisks above black columns refer to white columns; all others refer to black columns.

Unexpectedly, we observed an increase in the initiation time of ipsilateral forepaw stepping after 6-OHDA lesioning in one group and no beneficial effect of DBS in any of the groups. Moreover, we found an aggravating effect of DBS effect after 3 weeks that vanished 3 days after the cessation of DBS (Figure 7).

**Figure 8:**
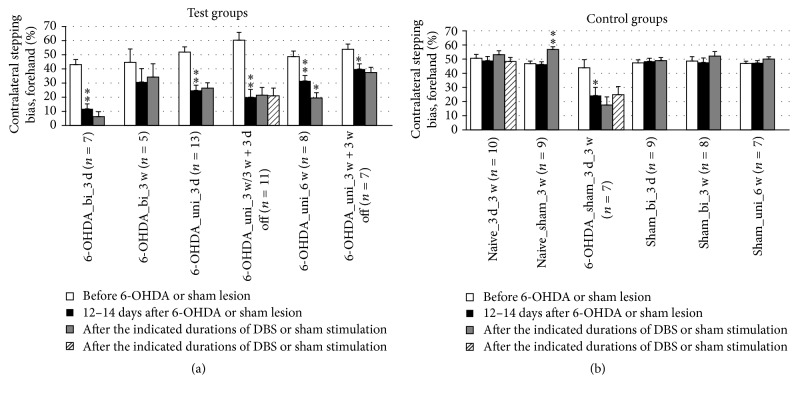
Short-term and long-term effects of DBS with uni- and bipolar electrodes on akinesia as measured by forced sidestepping of the forepaws in the forehand direction in the stepping tests. (a) and (b) refer to test groups and controls, respectively. Different column patterns indicate different times of behavioral testing; white: before 6-OHDA or sham lesion; black: 12–14 days after 6-OHDA or sham lesion; gray and hatched: after the durations of DBS or sham stimulation indicated in the group labels. For experimental details see Table 1. Significance levels are indicated with asterisks as follows: ^*∗∗*^*p* ≤ 0.01, ^*∗*^*p* ≤ 0.05, b (borderline): 0.05 < *p* ≤ 0.08 according to one-way ANOVA. Asterisks above black columns refer to white columns; all others refer to black columns.

**Figure 9:**
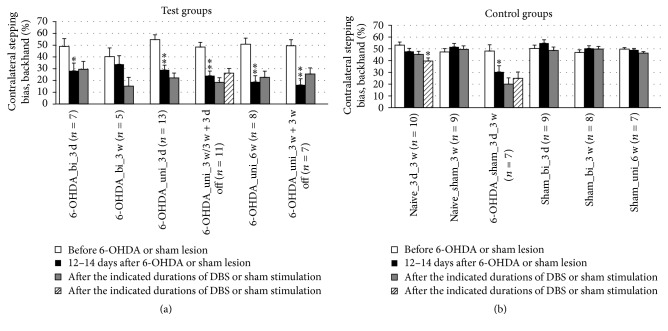
Short-term and long-term effects of DBS with uni- and bipolar electrodes on the akinesia of hemiparkinsonian rats as measured by forced sidestepping of the forepaws in the backhand direction in the stepping tests. (a) and (b) refer to test groups and controls, respectively. Different column patterns indicate different times of behavioral testing; white: before 6-OHDA or sham lesion; black: 12–14 days after 6-OHDA or sham lesion; gray and hatched: after the durations of DBS or sham stimulation indicated in the group labels. For experimental details see Table 1. Significance levels are indicated with asterisks as follows: ^*∗∗*^*p* ≤ 0.01, ^*∗*^*p* ≤ 0.05, b (borderline): 0.05 < *p* ≤ 0.08 according to one-way ANOVA. Asterisks above black columns refer to white columns; all others refer to black columns.

Impaired contralateral paw movement (contralateral bias) was determined based on the number of contralateral versus ipsilateral adjusting steps of the forepaws. A significant difference in the contralateral bias during forced sidestepping was found in only one case. The contralateral bias in the forehand direction worsened after 6 weeks of unipolar DBS (Figure 8). In the backhand direction, no significant effects of DBS were detected (Figure 9). Overall, the contralateral bias measured in the stepping test did not seem to be affected by DBS therapy (Table 2).

### 3.4. Sensorimotor Neglect

**Figure 10:**
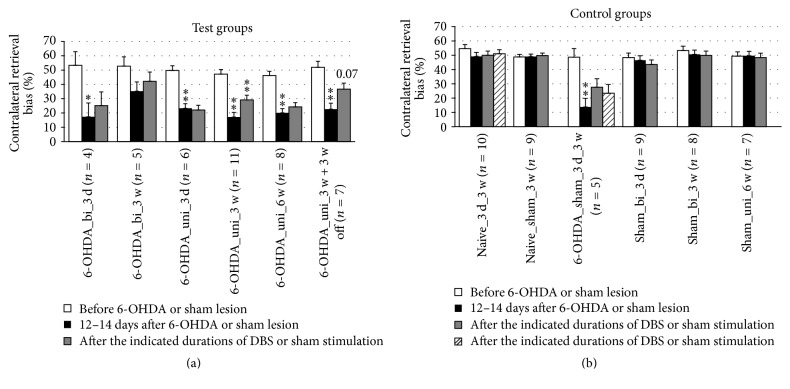
Short-term and long-term effects of DBS with uni- and bipolar electrodes on the sensorimotor neglect of hemiparkinsonian rats as measured by the corridor test. (a) and (b) refer to test groups and controls, respectively. Different column patterns indicate different times of behavioral testing; white: before 6-OHDA or sham lesion; black: 12–14 days after 6-OHDA or sham lesion; gray and hatched: after the durations of DBS or sham stimulation indicated in the group labels. For experimental details see Table 1. Significance levels are indicated with asterisks as follows: ^*∗∗*^*p* ≤ 0.01, ^*∗*^*p* ≤ 0.05, b (borderline): 0.05 < *p* ≤ 0.08 according to one-way ANOVA. Asterisks above black columns refer to white columns; all others refer to black columns.

In the corridor test, DBS reduced the amount of sensorimotor neglect when applied by unipolar electrodes for 3 weeks (Figure 10). The beneficial effect persisted for at least 3 weeks after the cessation of DBS, although with borderline significance. Interestingly, 6 weeks of continuous DBS did not demonstrate the same beneficial effect, and DBS with bipolar electrodes did not show any significant beneficial effects.

### 3.5. Anxiety

**Figure 11:**
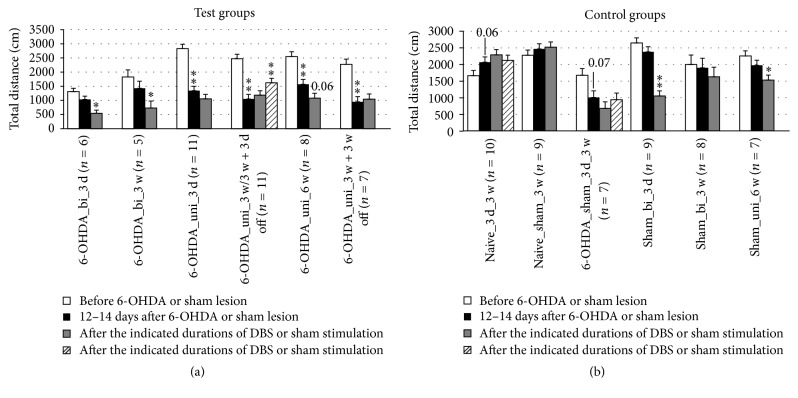
Short-term and long-term effects of DBS with uni- and bipolar electrodes on the locomotor activity of hemiparkinsonian rats as measured by the total migration distance in the open field. (a) and (b) refer to test groups and controls, respectively. Different column patterns indicate different times of behavioral testing; white: before 6-OHDA or sham lesion; black: 12–14 days after 6-OHDA or sham lesion; gray and hatched: after the durations of DBS or sham stimulation indicated in the group labels. For experimental details see Table 1. Significance levels are indicated with asterisks as follows: ^*∗∗*^*p* ≤ 0.01, ^*∗*^*p* ≤ 0.05, b (borderline): 0.05 < *p* ≤ 0.08 according to one-way ANOVA. Asterisks above black columns refer to white columns; all others refer to black columns.

**Figure 12:**
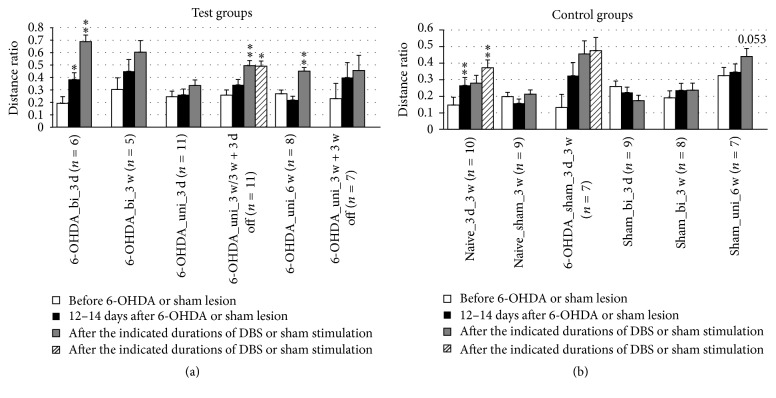
Short-term and long-term effects of DBS with uni- and bipolar electrodes on the anxiety-like behavior (b) of hemiparkinsonian rats as measured by the ratio: migration distance in the center/total migration in the open field. (a) and (b) refer to test groups and controls, respectively. Different column patterns indicate different times of behavioral testing; white: before 6-OHDA or sham lesion; black: 12–14 days after 6-OHDA or sham lesion; gray and hatched: after the durations of DBS or sham stimulation indicated in the group labels. For experimental details see Table 1. Significance levels are indicated with asterisks as follows: ^*∗∗*^*p* ≤ 0.01, ^*∗*^*p* ≤ 0.05, b (borderline): 0.05 < *p* ≤ 0.08 according to one-way ANOVA. Asterisks above black columns refer to white columns; all others refer to black columns.

The open field test provided information on locomotor activity and anxiety-like behavior. Although the total distance moved (Figure 11) was determined by both locomotor function and anxiety, the ratio of distances (central versus peripheral movement) predominantly reflects anxiety. In untreated naïve rats, the distance ratio generally increased with time, indicating a habituation effect, and sham DBS-treated naïve rats and DBS-treated sham-lesioned rats retained this behavior (Figure 12). However, the distance ratio significantly increased in the groups treated by DBS with unipolar electrodes after 3 or 6 weeks, but not after 3 days. In contrast, the distance ratio was increased in the groups treated by DBS with bipolar electrodes after 3 days, but not after 3 weeks. A minor increase (*p* = 0.053) in the distance ratio was also observed after 6 weeks of sham stimulation with unipolar electrodes.

Table 2 summarizes the test results for DBS-induced parameter changes.

## 4. Discussion

### 4.1. The Hemiparkinsonian Rat Model

The 6-OHDA-induced hemiparkinsonian rat model has been established for the study of therapeutic approaches for treating PD [11, 35, 49–51]. Although this model is known to reflect the major behavioral impairments that are characteristic of PD patients, animal studies are hampered by restrictions on free movement and/or invasive surgery and by short observation periods lasting from a few minutes [35, 49, 51–54] to a number of days [12, 24, 55–57]. To our knowledge, removable and reusable devices have been previously used by only Forni et al. [28].

Frequencies from 90 to 130 Hz are generally accepted as optimal to elicit the therapeutic effects of DBS in patients [52]. In this frequency range, the clinically observed benefits are maximal and a more normal activity pattern in the nuclei downstream is restored [22]. Recent findings in patients with advanced PD who became refractory to the common high-frequency stimulation have shown a restoration in the improvement of segmental and axial symptoms, gait disturbance, and levodopa-induced dyskinesia after the stimulation frequency was reduced to 60 Hz [58]. Here, we used a pulse frequency of 130 Hz in all experiments, even though this frequency was established for DBS in the much larger human brain.

In PD patients, unipolar stimulation is the preferred mode of DBS. So et al. [51] have also suggested using unipolar stimulation in the hemiparkinsonian rat model, although they did not find differences between the effects of uni- and bipolar stimulations in a drug-induced locomotor test. Our first experiments (up to 3 weeks) revealed greater beneficial effects with unipolar DBS than with bipolar DBS. For these reasons, only the unipolar experiments were extended out to 6 weeks (Figure 4).

### 4.2. The Outcome of the Different Behavioral Tests

To test the success of lesioning and test initial locomotor function, the classical drug-induced rotation assay was used. Pathological rotation is measured in response to the administration of either the dopamine (DA) receptor agonist apomorphine or the DA-releasing drug amphetamine [43, 44, 59, 60].

In partially lesioned animals, Hefti et al. [59] did not find apomorphine-induced rotation, whereas amphetamine induced a dose-dependent ipsilateral rotation. These authors observed a contralateral apomorphine-induced rotation only in severely lesioned animals, which is comparable to our results. These findings are in line with the results of Da Cunha et al. [60], who investigated the directions of rotation that were induced by either apomorphine or amphetamine in partially and severely lesioned animals. In severely 6-OHDA-lesioned mice, apomorphine-induced rotation was shown to be more informative than amphetamine-induced rotation in discriminating between the different degrees of lesions [61].

Interestingly, we found a reduction in apomorphine-induced rotation if DBS was applied for 3 days or 3 weeks in either the bipolar or unipolar modes, with the latter being more effective (Figure 5; Table 2). Apomorphine-induced rotation returned to pre-DBS levels after 6 weeks of continuous DBS or after 3 weeks of continuous DBS followed by 3 weeks without DBS. As discussed below, STN-DBS-induced therapeutic effects are not predicted to be reflected in the rate of apomorphine-induced rotation. Therefore, the reason for the reduction in apomorphine-induced rotation after 3 weeks of DBS was unclear. This effect was not surgery-induced because it did not occur in the 6-OHDA-lesioned sham-stimulated rats. Assuming that STN-DBS temporarily increases striatal DA turnover, as described by Meissner et al. [50], the hypersensitivity of DA receptors could be transiently reduced at a time scale of weeks. We assume that this effect was not permanent in our model because it is known that the dopaminergic neurons in the substantia nigra pars compacta (SNc) eventually degenerate leading to an almost complete lack of DA release in the striatum.

We believe that apomorphine-induced rotation is not an appropriate parameter for testing the beneficial effects of STN-DBS. Limitations of the apomorphine-induced rotation test have been previously demonstrated. Metz and Whishaw [62] have shown that the apomorphine-induced rotation rate did not correlate with spontaneous and skilled reaching or ladder rung walking tasks. In a study on apomorphine-induced rotation, Chang et al. [63] failed to demonstrate any effect of STN-DBS in 6-OHDA-induced hemiparkinsonian rats. They concluded that the apomorphine-induced imbalance of dopaminergic activation may not necessarily be improved by DBS. In contrast, STN-DBS has been shown to reduce or even reverse the direction of amphetamine-induced rotation in 6-OHDA-lesioned rats [11, 35, 49, 51]. Nevertheless, the amphetamine-induced rotation test has its limitations. Kirik et al. [64] showed that the test for the initiation time in stepping was a more sensitive metric than the amphetamine-induced rotation test. Because of these limitations, some authors have introduced new methods to evaluate the effect of DBS, such as an automated rotarod method for the drug-free quantitative evaluation of overall motor deficits [65].

Additionally, our results showing that a reduction of the initiation time of the contralateral forepaw was induced by DBS were not consistent with the results of the rotation test with unipolar stimulation. The shortest initiation times of the contralateral paw were observed 3 weeks after the cessation of DBS subsequent to 3 weeks of continuous DBS (Figure 6; Table 2). Our histological investigations showed that dopaminergic neurons were not regenerated in the substantia nigra (results not published). This suggests that the persistent DBS effects might be related to the neuronal plasticity in young rats. The increase of the initiation time of the ipsilateral paw after 6-OHDA lesion was much less than the increase on the contralateral side. However, this result did show that both hemispheres are affected by unilateral 6-OHDA lesion, as we have previously shown for the activation of astrocytes in the contralateral striatum after 6-OHDA lesion [66].

Locomotor activity changes detected in the open field test should be interpreted with caution as they may be influenced by various modifying factors, including habituation, the need for exploration, and anxiety effects. Indeed, we observed a habituation to the open field in naïve rats in both the total migration distance and in the anxiety parameter distance ratio. Lesions induced a reduction in locomotor activity, as measured by total migration distance. DBS induced a further decrease, even in sham-lesioned rats (Figure 11; Table 2). This additional decrease was reversed after the cessation of DBS and did not occur in sham-stimulated rats. We interpret the DBS-induced decrease in locomotor activity as a reduction in the health of the animals caused by the electrical stimulation. These results indicate that stimulation parameters have to be reconsidered in future experiments.

In PD patients, anxiety may result from not only the impairment of motor function but also dysfunction of the STN. Experiments with bilaterally STN-lesioned rats in the elevated plus maze test also suggest such a connection [67]. Here, we assessed anxiety-like behavior using the open field parameter “ratio of migration distance in the center to total migration.” This parameter quantifies the balance between the need of the animal to explore their environment with the need to be cautious, which prevents them from exploring the unprotected center of a brightly lit open field box. In our setting, naïve rats became more curious and less anxious over time due to habituation. Likewise, the decrease in anxiety-like behavior by 6-OHDA-lesioned rats can be explained by habituation to the open field (Figure 12). Based on these results, the increasing distance ratio observed after DBS may not be an effect of DBS but rather an effect of habituation. In contrast, the distance ratio of sham-lesioned rats and sham-stimulated naïve rats (i.e., healthy rats with disconnected electrodes) remained at their initial levels. The reduced activity of the rats after electrode implantation suggests adverse effects of the surgery itself and a treatment-related reduction in the rats' health.

The corridor test was originally established to detect lateralized sensorimotor integration [61]. It has been successfully applied to demonstrate the feasibility of the thalamic center-median parafascicular nucleus as a target for DBS in 6-OHDA-lesioned rats [55, 68]. In our study, 3 weeks of STN-DBS with unipolar electrodes reduced sensorimotor neglect. After the cessation of DBS, this effect persisted with borderline significance for at least 3 more weeks (Figure 10). Six weeks of continuous DBS did not have the same beneficial effect.

These findings raise questions about whether different mechanisms are responsible for the observed effects of acute and chronic DBS, as well as about the persistent effects on locomotor and sensorimotor functions. One possible reason for these differences may be the development of an insensitivity toward DBS, reflecting changes in the basal ganglia network [22]. In addition, readjustment of the stimulation parameters may be necessary in chronic DBS, as is common in clinical practice, to compensate for the increasing impedance caused by the development of adventitia tissue at the electrode-tissue interface [69].

Here, we propose that tests of spontaneous locomotion, such as the stepping test, are more relevant for detecting the beneficial effects of DBS and provide different information than the apomorphine-induced rotation test. However, this conclusion does not necessarily apply to the amphetamine-induced rotation test because of the different mechanisms of these two rotation tests (see Appendix).

Our results suggest that persistent DBS effects in 6-OHDA-lesioned neuronal networks may be the result of the protection or regeneration of part of the physiological function of these networks in relation to locomotor activity in the absence of dopaminergic neurons. Alternatively, persistent effects of DBS could arise from DBS-induced effects that mimic a permanent lesion of the STN, for example, by space-consuming effects of the developing adventitia. Such mechanisms may explain our findings of persistent DBS effects on the initiation time of the contralateral forepaw in the stepping test (Figure 6) and, though with borderline significance, on sensorimotor neglect in the corridor test (Figure 10). A DBS-induced “mimicked” STN lesion would be in agreement with the lack of beneficial effects demonstrated in the open field behavior.

**Figure 13:**
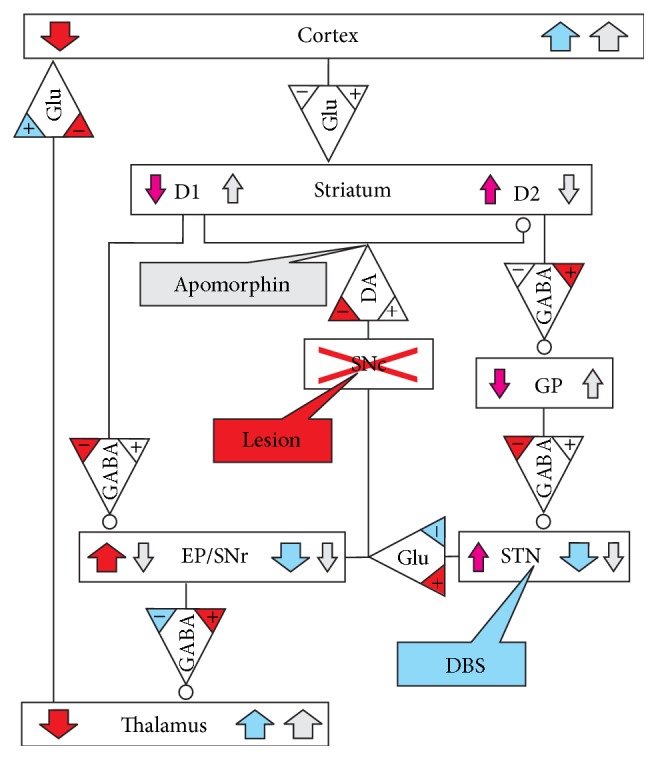
Simplified scheme of the lesioned hemisphere describing the effects of the lesion (red), DBS (blue), and apomorphine administration (light gray) on neurotransmitter release and on the activity (colored arrows) of different brain areas. Induced alterations in receptor numbers or sensitivities are not depicted; for explanation see Appendix. The brain areas are given in the boxes designated by SNc, GP, STN, and EP/SNr. The neurotransmitters glutamate (Glu), DA, and GABA are designated by triangular boxes pointing toward the affected brain areas that may be either excited (direct line input) or downregulated (input with circle). D1 and D2 stand for the dopaminergic receptors in the striatum, which are excited or inhibited by DA or apomorphine, respectively. Rectangular text balloons mark the input sites of lesioning, DBS, and apomorphine. The colored “+” and “−“ signs in the neurotransmitter triangles designate the effects of lesioning (red) and DBS (blue) on transmitter release. Color-coding was not attempted for the effects of apomorphine on transmitter release.

The different brain states and resulting behavioral effects are considered in Appendix.  Figure 13 illustrates four different brain states in a single scheme of the lesioned hemisphere in the hemiparkinsonian rat: the healthy brain, the effects of a lesion, DBS after lesioning, and apomorphine administration after lesioning.

### 4.3. Effects at the Molecular and Receptor Levels

Based on previous studies, apparently contradictory results have been obtained at both the molecular and receptor levels. The DBS-related decreases in the levels of extracellular DA and its metabolites in the dorsal part of the striatum described by Walker et al. [70] are in line with a decreased concentration of the DA metabolite DOPAC (3-4-dihydroxyphenylacetic acid) in the extracellular fluid of the striatum found by Yamamoto et al. [71]. In contrast, He et al. [72] described a DBS-induced increase in the extracellular striatal DA concentration. Recently, GABAergic activation by chronic DBS has been shown to be responsible for the compensation of motor asymmetries in hemiparkinsonian rats [73].

At the level of the receptors, missing neurotransmitter inputs are believed to induce a compensatory upregulation of receptor numbers or sensitivity. According to this view, a lesion-induced reduction in glutamate release to the cortex and the striatum resulting from alterations in activity along the striatum-D1 receptor-EP/SNr-thalamus pathway will result in an upregulation in glutamate receptors in the cortex and striatum. However, DBS after lesioning was found to reverse the increased striatal glutamate receptor numbers [57] and to increase the number of D1 receptors, which probably improves motor symptoms in PD patients [74]. At the same time, DBS decreases the number of D2/D3 receptors in the nucleus accumbens of rats, which may contribute to adverse DBS-induced neuropsychiatric side effects, such as apathy [74].

Most biochemical studies have been conducted under acute or subchronic (up to 7 days) STN-DBS. However, a deeper insight into the DBS-mechanisms and its long-term or persistent effects (≥6 weeks) require animal models that are suitable for combining biochemical, electrophysiological, optical microscopy, and other imaging methods, along with behavioral testing.

## 5. Conclusion and Outlook

To our knowledge, we present the first behavioral investigation in freely moving rats with chronic instrumentation for up to 6 weeks, which allowed the animals to adapt to the instrumentation and allowed us to conduct comparative behavioral tests at different times under acute DBS conditions and after the cessation of DBS. In our setup, we found unipolar stimulation to be more efficient for achieving several beneficial long-term DBS effects. In our tests of behavioral changes, the stepping and corridor tests were the most appropriate for the evaluation of DBS-induced locomotor and sensorimotor improvements. When DBS was stopped after 3 weeks, some effects persisted for at least 3 more weeks, such as the reduction of initiation time of the contralateral paw in the stepping test and the slight reduction of the contralateral bias in the corridor test. In contrast, performance in the apomorphine-induced rotation test showed no improvement after 6 weeks. Our findings may indicate a regeneration of neuronal circuits in the absence of dopaminergic neurons. This would make apomorphine-induced rotation a suitable test to determine the long-term success of 6-OHDA lesioning but not a very informative test for determining the beneficial effects of DBS. In interpreting anxiety-like behaviors, researchers must consider habituation effects in relation to the durations between test repetitions in both sham and experimental animals.

The determination of very fast reaction times was difficult. To improve the statistical power of these tests, a larger sample size should be combined with video detection of reaction times. Our model can be considered a versatile platform that allows for the independent testing of separate elements, such as electrodes and counter-electrodes. Relatively simple modifications to our model will allow for the testing of unexplored target regions in other neurodegenerative disorders.

In addition, various electrical parameters can be tested, such as stimulation frequency and signal shape. To our knowledge, no systematic investigations have been conducted on whether the frequencies applied to humans are suitable for use with much smaller animals. We believe that this topic needs further investigation, taking into account allometric effects for organisms with various brain sizes. Our results may help in developing a reduced set of test parameters to facilitate this research.

Major problem remains to be elucidated about the mechanism by which DBS acts. Although 6-OHDA lesioning induces PD-like symptoms, the long-term DBS effects in our model may be a result of the emergence of new or a strengthening of existing neuronal circuits that compensate for the absence of dopamine in the brains of young rats. This outcome may suggest that the DBS-related locomotor and sensorimotor improvements, with no detectable improvements in the results of the rotation test, indicate DBS effects in the activation of neuronal substitute circuits. If this hypothesis is supported by future research, investigations of the effects of stimulation may be helpful in other areas, such as stroke research.

## Figures and Tables

**Table 1:** Experimental design. The number of rats refers to the group sizes at the time of the apomorphine-induced rotation tests.

Group name	6-OHDA lesion	Electrode	DBS duration	Number of animals
Naive_3 d/3 w	−	—	—	10
Naive_sham_3 w	−	Bipolar	—	9
6-OHDA_sham_3 d/3 w	+	Bipolar	—	7
Sham_bi_3 d	Sham	Bipolar	3 days	9
Sham_bi_3 w	Sham	Bipolar	3 weeks	10
Sham_uni_6 w	Sham	Unipolar	6 weeks	7
6-OHDA_bi_3 d	+	Bipolar	3 days	7
6-OHDA_bi_3 w	+	Bipolar	3 weeks	5
6-OHDA_uni_3 d	+	Unipolar	3 days	13
6-OHDA_uni_3 w/3 w + 3 d off	+	Unipolar	3 weeks	11
6-OHDA_uni_6 w	+	Unipolar	6 weeks	7
6-OHDA_uni_3 w + 3 w off	+	Unipolar	3 weeks	8

**Table 2:** Summary of test results. The improvement and worsening of lesion-induced parkinsonian symptoms by DBS are marked by (+) and (−), respectively. Borderline changes (0.05 < *p* ≤ 0.08) are marked by (b); parameters with no detectable DBS effects are marked (0); n.d. stands for “not determined.” The results marked with an asterisk could not be interpreted in terms of the therapeutic DBS effects.

Group name	Rotation test	Stepping test initiation time	Stepping test contralateral bias		Corridor test	Open-field test	
		Contralateral paw	Forehand	Backhand		Total distance	Distance ratio^*∗*^
6-OHDA_bi_3 d	b+	+	0	0	0	—	Up
6-OHDA_bi_3 w	b+	b+	0	0	0	—	0
6-OHDA_uni_ 3 d	+	b−	0	0	0	—	0
6-OHDA_uni_ 3 w	+	0	0	0	+	0	Up
6-OHDA_uni_ 3 w + 3 d off	0	0	0	0	n.d.	+	Up
6-OHDA_uni_ 6 w	0	b+	—	0	0	b−	Up
6-OHDA_uni_ 3 w + 3 w off	0	+	0	0	b+	0	0

## Appendix

### A Simplified Scheme of the Lesioned Hemisphere in the Hemiparkinsonian Rat

Figure 13 presents a simplified scheme of the lesioned hemisphere, illustrating different brain states, which are immediately induced by alterations in neurotransmitter release and in the activity of different brain areas. Long-term alterations in receptor numbers or sensitivities are not depicted. It should be noted that the scheme can attempt only a qualitative description of the separate effects. The interplay of the lesioned hemisphere with its nonlesioned counterpart must be considered to explain the overall effect, for example, the effect of amphetamine administration. The rotation effects may be explained by the assumption that the induced hyperactivation of the cortex of one hemisphere leads to a general pattern of muscle activation contralateral to the hyperactive side, which would result in a bending of the body toward this side and, subsequently, to rotation contralateral to the hyperactive hemisphere.

The depicted effects will, in principle, also apply when one of the successive treatments is omitted. Thus, the scheme allows for predicting multiple scenarios, such as the effects of DBS without a lesion or apomorphine administration without DBS. Nevertheless, the actual magnitudes of the combined effects on the activity of the various brain areas may vary significantly, leading to different individual responses. Short summaries of the different states illustrated by Figure 13 are given below.

*Healthy Brain*. No colors or arrows, which designate deviations from the normal brain activity, apply.

*Lesion Effect*. Obliteration of the SNc (red cross) stops DA input for D1 and D2 receptors in the striatum. Along the D1 pathway, the reduced GABAergic inhibition results in an increased activity of the entopeduncular nucleus/substantia nigra pars reticulata (EP/SNr). Along the D2 pathway, the effect on the globus pallidus (GP) is inverse. The increased GABAergic inhibition of the GP leads to a reduced inhibition of the STN, resulting in an increased activity of the EP/SNr in line with the effect on the D1 pathway (note that reduced arrow widths symbolize the parallel D1 and D2 signaling pathways to the EP/SNr). The simultaneous effects of the D1- and D2-signaling pathways result in an increase in EP/SNr activity, which leads to an increased inhibition of the thalamus and a subsequent reduction in the activity of the cortex of the lesioned hemisphere.

*Apomorphine Effect (Induced Rotation) after Lesion*. The missing DA input to the striatum after SNc obliteration leads to an oversensitization of the D1 and D2 receptors to apomorphine, a DA agonist, in the striatum of the lesioned side. After systemic administration of apomorphine, both the D1- and D2-mediated effects (gray arrows) lead to a downregulation of the EP/SNr and, consequently, a stronger output from the thalamus to the cortex of the ipsilateral hemisphere compared to the contralateral hemisphere, where D1 and D2 receptors retain normal sensitivity. Overactivity of the ipsilateral cortex increases muscle tension on the contralateral side of the body, resulting in a bending that leads to contralateral rotation.

*DBS Effect*. DBS reduces the hyperactivity of the STN, which results in a reduced activity of the EP/SNr. In an ideal case (as indicated by the width of the blue arrow in the STN box), this reduced activity may normalize the inhibition of the thalamus and consequently the cortex activity. This outcome requires that STN-DBS can overcompensate the effect of the lesion along the EP/SNr pathway. If so, unilateral DBS in nonlesioned animals should overactivate the thalamus and the cortex in the stimulated hemisphere. Indeed, like apomorphine in lesioned animals, DBS in nonlesioned rats has been shown to induce contralateral rotation [75]. In lesioned animals, DBS is unable to reverse the hypersensitivity of the D1 and D2 receptors and thus has no effect on apomorphine-induced rotation. The concordant DBS and apomorphine effects prevent the detection of therapeutic DBS effects in apomorphine-induced rotation tests.

*Amphetamine Effect (Induced Rotation) after Lesion*. Amphetamine induces an increased DA release and inhibits its reuptake. In 6-OHDA-lesioned rats, amphetamine effects can occur in only the contralateral hemisphere because the dopaminergic neurons are degenerated in the ipsilateral SNc. Systemic amphetamine administration hyperactivates the contralateral cortex, resulting in ipsilateral rotation. DBS should reduce the asymmetry in the cortex activities of the hemispheres by activating the cortex of the lesioned (ipsilateral) hemisphere. Indeed, a reduction or even reversion of the amphetamine-induced rotation by STN-DBS was found in 6-OHDA-lesioned rats [11].

These considerations suggest a higher relevance of amphetamine-induced rotation tests for assessing therapeutic DBS effects, although apomorphine-induced rotation tests are useful for determining the success and degree of lesion induction.
